# Regulated proteolysis of the alternative sigma factor SigX in *Streptococcus mutans*: implication in the escape from competence

**DOI:** 10.1186/1471-2180-14-183

**Published:** 2014-07-09

**Authors:** Gaofeng Dong, Xiao-Lin Tian, Zubelda A Gomez, Yung-Hua Li

**Affiliations:** 1 Department of Applied Oral Sciences, Faculty of Dentistry, Dalhousie University, 5981 University Avenue, Halifax, Nova Scotia B3H 1 W2, Canada; 2 Department of Microbiology and Immunology, Faculty of Medicine, Dalhousie University, 5850 College Street, Halifax, Nova Scotia B3H 4R2, Canada

**Keywords:** Regulated proteolysis, Adaptor protein, Competence, *Streptococcus mutans*

## Abstract

**Background:**

SigX (σ^X^), the alternative sigma factor of *Streptococcus mutans,* is the key regulator for transcriptional activation of late competence genes essential for taking up exogenous DNA. Recent studies reveal that adaptor protein MecA and the protease ClpC act as negative regulators of competence by a mechanism that involves MecA-mediated proteolysis of SigX by the ClpC in *S. mutans*. However, the molecular detail how MecA and ClpC negatively regulate competence in this species remains to be determined. Here, we provide evidence that adaptor protein MecA targets SigX for degradation by the protease complex ClpC/ClpP when *S. mutans* is grown in a complex medium.

**Results:**

By analyzing the cellular levels of SigX, we demonstrate that the synthesis of SigX is transiently induced by competence-stimulating peptide (CSP), but the SigX is rapidly degraded during the escape from competence. A deletion of MecA, ClpC or ClpP results in the cellular accumulation of SigX and a prolonged competence state, while an overexpression of MecA enhances proteolysis of SigX and accelerates the escape from competence. *In vitro* protein-protein interaction assays confirm that MecA interacts with SigX via its *N*-terminal domain (NTD_1–82_) and with ClpC via its *C*-terminal domain (CTD_123–240_). Such an interaction mediates the formation of a ternary SigX-MecA-ClpC complex, triggering the ATP-dependent degradation of SigX in the presence of ClpP. A deletion of the *N*-terminal or *C*-terminal domain of MecA abolishes its binding to SigX or ClpC. We have also found that MecA-regulated proteolysis of SigX appears to be ineffective when *S. mutans* is grown in a chemically defined medium (CDM), suggesting the possibility that an unknown mechanism may be involved in negative regulation of MecA-mediated proteolysis of SigX under this condition.

**Conclusion:**

Adaptor protein MecA in *S. mutans* plays a crucial role in recognizing and targeting SigX for degradation by the protease ClpC/ClpP. Thus, MecA actually acts as an anti-sigma factor to regulate the stability of SigX during competence development.

## Background

Regulated proteolysis by ATP-dependent proteases is widely distributed in both prokaryotic and eukaryotic cells, and is an important mechanism for the timely turnover of many intracellular proteins [1-3]. Representative examples of the proteases in bacteria include the Clp superfamily proteases, such as ClpAP, ClpCP, ClpXP, ClpB, ClpE and ClpL, which play important roles in regulating various cellular functions, such as growth, sporulation, competence development, virulence, and general stress responses [4-6]. These proteases ensure the welfare of a cell by removing misfolded and regulatory proteins, whose degradation may be vital to the control of signaling cascades, metabolic or developmental pathways or cell fate under a specific condition [1-6]. For degradation of right proteins at right times, eukaryotic cells use the ubiquitin-tagging machinery to recognize proteins and mark them for degradation by the proteasome [1-3]. However, most bacteria carry out selective degradation of proteins by using adaptors that provide the ability to recognize specific substrates for degradation by the proteases [7-10]. Several adaptor proteins have been identified and characterized in both Gram-negative and Gram-positive bacteria, such as SspB and ClpS in *E. coli*, and MecA, YjbH, YpbH and McsB in *B. subtilis*[7-11]. These adaptor proteins provide a way to modulate the substrate specificity for protein degradation and play important roles in quality control of cellular proteins and their abundance. Therefore, control of the availability and activity of adaptor proteins may be essential for bacteria in response to changing environments.

Competence development is a transient physiological state or X state in which the bacteria take up exogenous DNA from the environment [12-14]. In streptococci, the key step of competence induction is the transcriptional activation of *comX* that encodes an alternative sigma factor SigX (σ^X^) controlling expression of late competence genes essential for DNA uptake and recombination [14-16]. It is now known that two quorum-sensing signaling systems, the ComCDE and ComRS, are involved in the regulation of genetic competence in *S. mutans*[17-19]. When grown in a complex medium, *S. mutans* secretes, senses and responds to a competence-stimulating peptide or CSP through the ComCDE pathway [20]. CSP can be detected by the ComD histidine kinase of a two-component system ComDE, leading to autophosphorylation of its cognate response regulator ComE that in turn activates transcription of an array of the target genes [20-22]. The ComCDE system coordinates the production of several nonlantibiotic bacteriocins (mutacins) and also strongly induces expression of *comX,* resulting in competence activation in a subpopulation [21-23]. However, the pathway transmitting this signal from the ComE response regulator to *comX* is unclear, since the ComE binding site is not identified in the promoter region of *comX* in *S. mutans*[22,24]. This missing link has been recently filled by identification of the type II ComRS signaling system that proximally controls transcription of *comX* in *S. mutans*[18]. When grown in a chemically defined medium (CDM), *S. mutans* secretes a *sigX*-inducing peptide or XIP, its precursor encoded by *comS*[25-27]. Once internalized by an oligopeptide permease transporter, Opp, the XIP interacts with the ComR regulator to form a ComR/XIP complex that drives transcription of *comX*, triggering the competence cascade [25-27]. Competence activation through the ComRS signaling system is much stronger, nearly involving the entire population [25-27]. Thus, *S. mutans* can finely regulates transcription of *comX* for competence through a regulatory network that receives and responds to two signaling peptides, dependently on nutrient conditions in the environment.

Besides the transcriptional control of *comX*, SigX protein is also the target of posttranslational regulation in streptococci. In *S. penumoniae*, SigX is positively regulated by ComW but is negatively controlled by the protease ClpE/ClpP [28-30]. Since ComW is labile to the protease ClpC/ClpP, proteolysis of ComW by the ClpC/ClpP can also negatively affect the activity and stability of SigX in *S. pneumoniae*[28-30]. Posttranslational regulation of competence is well documented in *B. subtilis*, in which the master regulator ComK that triggers competence is sequestered in a ComK-MecA-ClpC complex [31-33]. The ComK is activated when it is released from the complex by a small protein ComS [32]. Recently, a similar mechanism has been identified in *S. mutans* and *S. thermophilus*, in which MecA and ClpC act as negative regulators of genetic competence by a mechanism that requires the presence of a functional SigX [34-36]. MecA in these species interacts with both SigX and ClpC, mediating the formation of a ternary SigX-MecA-ClpC complex that sequesters the SigX activity [34,35]. These findings have led us to hypothesize that adaptor protein MecA may target SigX for degradation by the ClpC/ClpP protease*.* In this study, we set forth the experiments to test this hypothesis by investigating MecA-mediated proteolysis of SigX and the impacts on competence regulation in *S. mutans*.

## Results

### The cellular level and stability of SigX during competence induction by CSP

**Figure 1:**
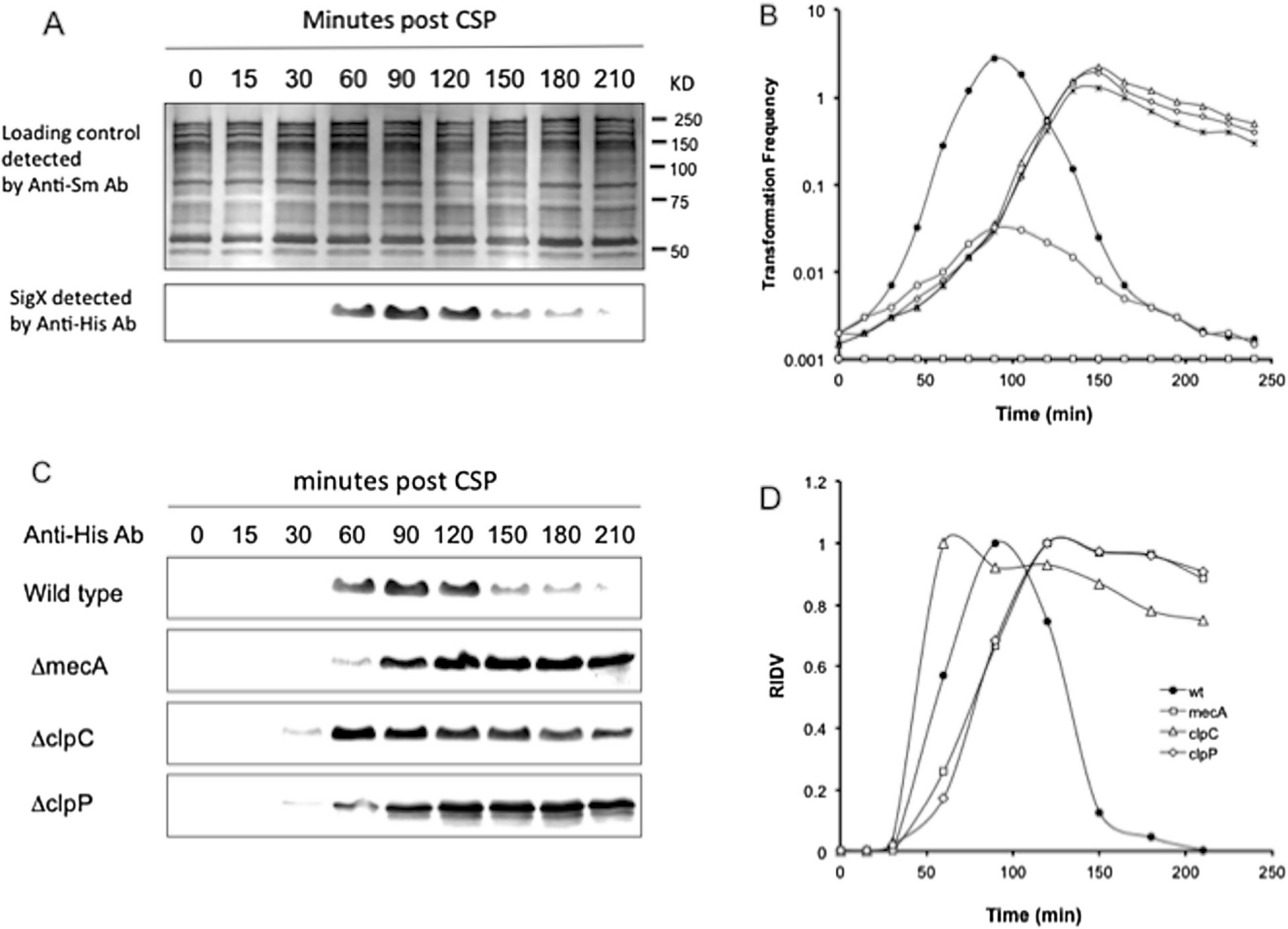
**The cellular levels of SigX in *S. mutans *during competence induction by CSP and the effects of *mecA*, *clpC *or *clpP *deletion on the stability of SigX and competence. A**. Western blot analysis of the cellular levels of SigX in *S. mutans* strain XT-His1 (wt) using the anti-His antibody. The total protein levels from the cell lysates of this strain are included as an example of protein loading controls detected by Western blotting using the anti-*S. mutans* antibody. **B**. Competence induction by CSP and the effects of *mecA*, *clpC* or *clpP* deletion on the transformation efficiency of *S. mutans* UA159 (wt, black circle), XT-D1 (∆*comX*, open squares), XT-D4 (∆*mecA*, open diamonds), XT-D7 (∆*clpC*, open triangles), XT-D8 (∆*clpP*, black stars) and GF-Ox (MecA-Ox, open circles). **C**. Western blot analysis of the effects of *mecA*, *clpC* or *clpP* deletion on the cellular levels of SigX in *S. mutans* strains XT-His1 (wt), XT-His2 (∆*mecA*), XT-His3 (∆*clpC*) and GF-His1 (∆*clpP*). **D**. The protein bands representing the cellular levels of SigX in these strains were scanned and the intensities were converted as relative integrated density values (RIDV), which were normalized to a maximum value of 1.0 for each strain.

Previous studies showed that CSP induced a transient competence state or X state that allowed a subpopulation to take up transforming DNA when *S. mutans* was grown in a complex medium, the growth condition that is sub-permissive for competence development [17-22]. The X state normally maintained for 40–70 min and then shut off, suggesting that the SigX activity was unstable following transient competence activation. To confirm this observation, we examined the cellular level of SigX in a *S. mutans* wild type background strain XT-His1 (wt) grown in THYE for competence induction by CSP. This strain that carried a shuttle vector pSigX-His allowed detection of His-tagged SigX in the crude cell lysates by Western blotting. Without addition of CSP (T_0_), SigX was undetectable until about 60 min following addition of CSP a relatively high level of SigX was detected in the cell lysates (Figure 1A). This level of SigX remained for about 60 min and then rapidly declined to the levels nearly undetectable. However, the total protein levels in the cell lysates were relatively stable over times, particularly a 53-kDa protein, as indicated in the protein loading controls detected by Western blotting using the anti-*S. mutans* (serotype C) antibody. Consistent with the cellular levels of SigX, *S. mutans* UA159 (wt) showed relatively high but transient levels of transformation in response to CSP (Figure 1B). In contrast, the *comX* deletion mutant (Δ*comX*; negative control) completely lost its transformability under the same condition. The results suggest that the synthesis of SigX is induced during competence induction by CSP, but SigX is quickly degraded during the escape from competence.

### Inactivation of MecA, ClpC or ClpP results in the cellular accumulation of SigX

Next, we examined the effects of inactivation of MecA, ClpC or ClpP on the cellular levels of SigX, since both MecA and ClpC were found to negatively affect the stability of SigX in THYE [34]. Three mutant background strains, XT-His2 (Δ*mecA*), XT-His3 (Δ*clpC*), GF-His1 (Δc*lpP*) and one wild-type strain XT-His1 that all carried a shuttle vector pSigX-His were used for Western blot analysis of cellular levels of SigX. The results showed that SigX was initially undetectable in all the strains without addition of CSP. Approximately at 60 min post CSP, all the mutant strains produced considerable levels of SigX (Figure 1C), which were similar to that in wild-type control XT-His1 (wt). However, all the mutant background strains retained relatively high levels of SigX throughout the experiments, suggesting an increased stability of SigX in the MecA-, ClpC- and ClpP-deficient strains. It was noted that the SigX appeared to accumulate even earlier (at 30 min) in ΔclpC and ΔclpP mutants than ΔmecA mutant. We considered this might be a variation of samplings during preparation of the crude cell lysates, since these strains did not grow at the same rate following addition of CSP. To compare the cellular levels of SigX over time, we scanned the protein bands and converted their intensities as relative integrated density values (RIDV), with a maximum value of 1.0 for each strain (Figure 1D). The wild type strain XT-His1 (wt) showed the highest intensity at 90 minutes post CSP and this intensity rapidly decreased to a very low level. In contrast, all the mutants remained relatively high levels of the intensities throughout the experiments, suggesting that deletion of *mecA, clpC* or *clpP* negatively affect degradation of SigX. Consistent with the cellular levels of SigX, these mutant strains showed a prolonged competence state to take up transforming DNA under this growth condition (Figure 1B). The cellular accumulation of SigX in these strains may prevent the timely escaping of the cells from competence. The results suggest that MecA, ClpC and ClpP are required for regulated proteolysis of SigX when *S. mutans* is grown in THYE medium.

### Overexpression of MecA enhances proteolysis of SigX and accelerates the escape from competence

**Figure 2:**
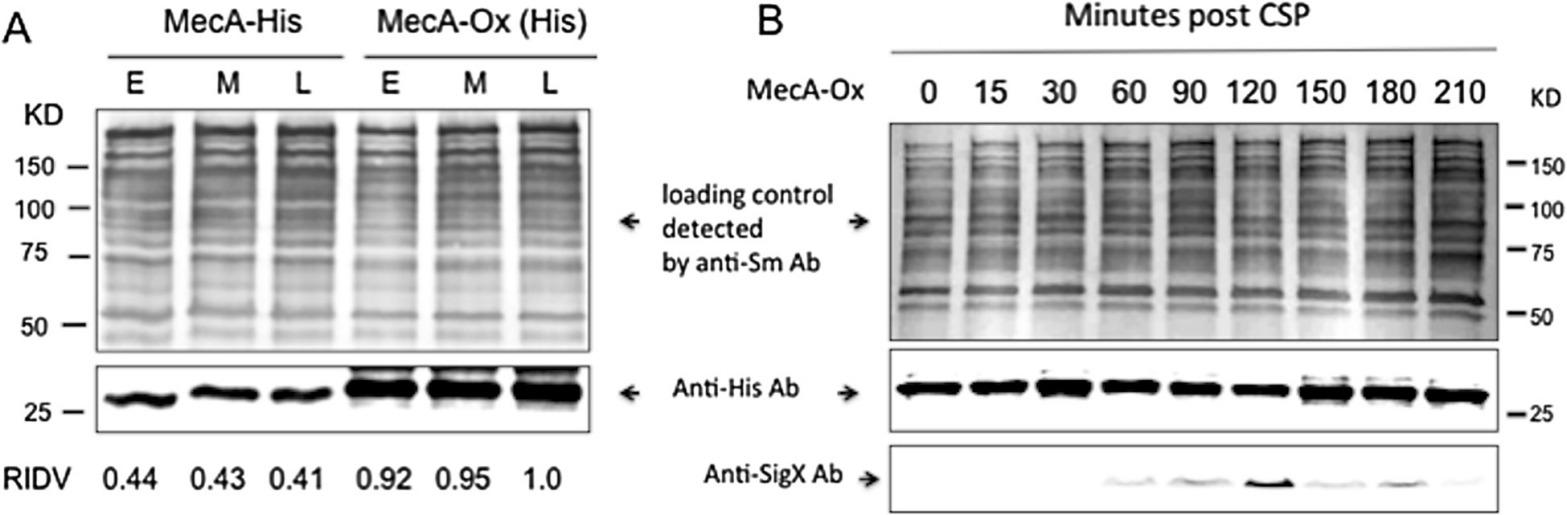
**Constitutive expression of MecA and its effect on the cellular levels of SigX. A**. Western blot analysis of cellular levels of MecA in *S. mutans* strain GF-His2 (MecA-His) and strain GF-Ox (MecA-Ox) that constitutively expressed MecA. The samples were taken from the cultures of these strains grown during the early- (E), mid- (M) and late- (L) exponential phases to prepare the crude cell lysates. The cellular MecA of these strains were detected by Western blotting using the anti-His antibody. The intensities of the bands were scanned and converted as relative integrated density values (RIDV) for comparison. The total protein loading controls from the cell lysates of these strains were detected using the anti-*S. mutans* antibody. **B**. The effect of constitutively expressed MecA on the cellular levels of SigX in GF-Ox (MecA-Ox) grown in THYE in response to CSP. The cellular MecA was detected by Western blotting using the anti-His antibody, while the cellular SigX in the same strain was detected using the anti-SigX antibody.

To further investigate the effect of MecA on the stability of SigX, we constructed a constitutively expressed MecA strain, GF-Ox (MecA-Ox), which expressed MecA under the control of a constitutively expressed promoter of *ldh*, the gene encoding lactate dehydrogenase in *S. mutans*. In addition, this strain allowed detection of MecA protein by Western blotting using the anti-His antibody, since a His-tag was added to the end of *mecA*-coding sequence just before the stop codon. Our results confirmed that strain MecA-Ox constitutively expressed MecA when grown in THYE, independently on the growth phases. Based on the relative integrated density values (RIDV), this strain (MecA-Ox) produced as much as two-fold MecA protein when compared with that of strain GF-His2 (MecA-His) grown under the same condition (Figure 2A), suggesting that strain MecA-Ox overexpressed MecA under the conditions tested. It was noted from the Western blot that MecA-Ox showed an extra band that slightly bigger than the MecA. The feature of this extra band was unclear. One possibility was that over-expressed MecA might also bind to a small, unknown protein or peptide in *S. mutans*. Alternatively, this band might be a non-specific reaction. We then began to determine the effect of an overexpressed MecA on the cellular levels of SigX in response to CSP. The results revealed that the overexpression of MecA negatively affected the cellular levels of SigX in this strain, since addition of CSP induced very low levels of SigX, as detected by Western blotting using the anti-SigX antibody (Figure 2B). Consistent with this result, the constitutive expression of MecA resulted in hundred-fold reduction in transformation efficiency (Figure 1B). The results suggest that the overexpressed MecA enhances MecA-mediated proteolysis of SigX, accelerating the escape from competence.

### Regulated proteolysis of SigX is ineffective when *S. mutans* is grown in CDM

**Figure 3:**
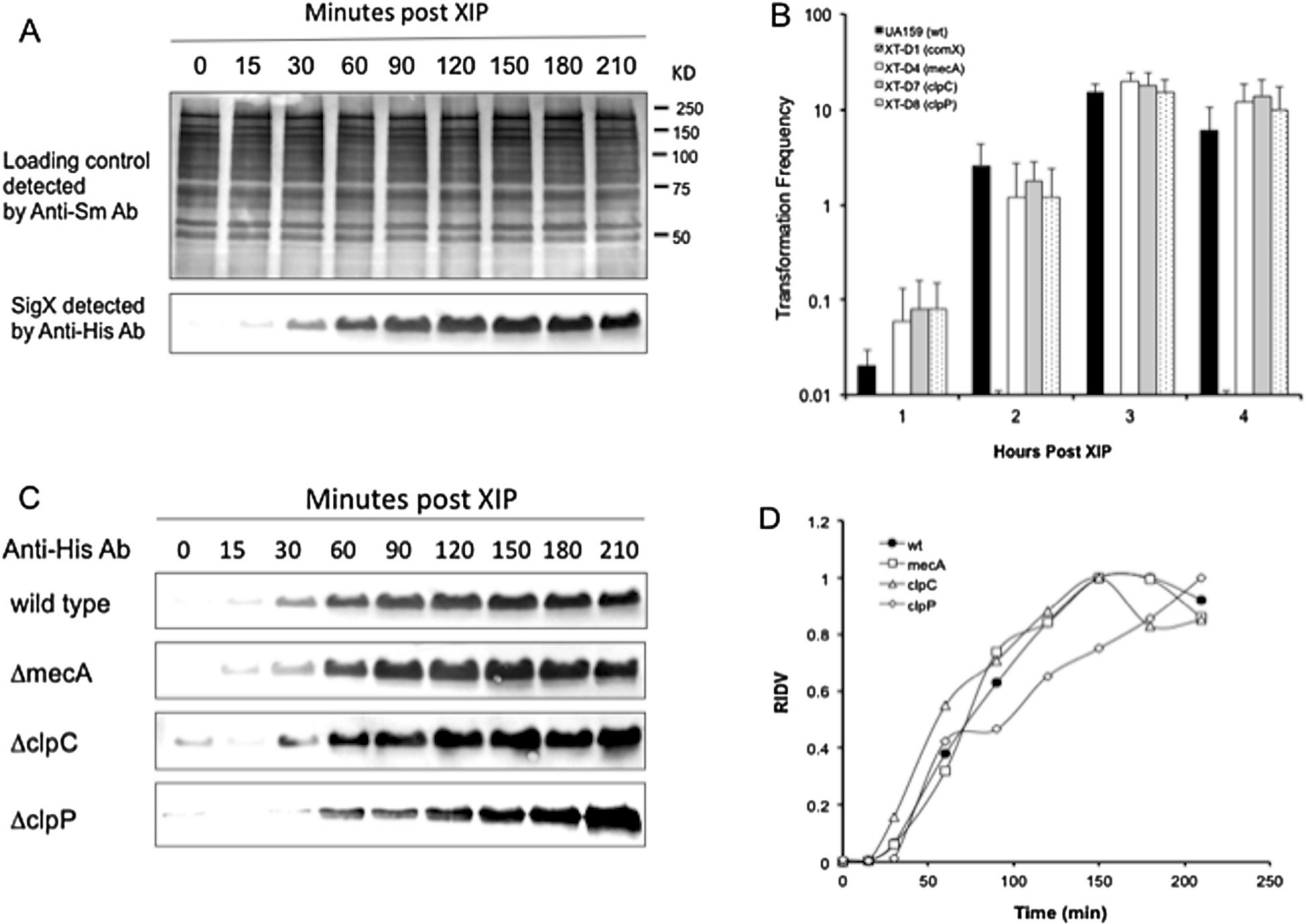
**The cellular levels of SigX during competence induction by XIP in CDM and the effects of *mecA*, *clpC *or *clpP *deletion on the stability of SigX and competence. A**. Western blot analysis of the cellular levels of SigX in strain XT-His1 (wt) by the anti-His antibody. The protein loading controls this strain were detected by Western blotting using the anti-*S. mutans* antibody. **B**. The effects of *mecA*, *clpC* or *clpP* deletion on the transformation efficiency of *S. mutans* strains UA159 (wt), XT-D1 (∆*comX*), XT-D4 (∆*mecA*), XT-D7 (∆*clpC*) and XT-D8 (∆*clpP*). **C**. Western blot analysis of the effects of *mecA*, *clpC* or *clpP* deletion on the cellular levels of SigX in strains XT-His1 (wt), XT-His2 (∆*mecA*), XT-His3 (∆*clpC*) and GF-His1 (∆*clpP*) using the anti-His antibody. **D**. The protein bands representing the cellular levels of SigX in these strains were scanned and the intensities of the bands were converted as the relative integrated density values (RIDV), which were normalized to a maximum value of 1.0 for each strain.

Recent studies show that when *S. mutans* is grown in a chemically defined medium (CDM), competence induction through the ComRS signaling system is active for hours in response to XIP [18,25,26]. These studies suggest that *S. mutans* activates competence differently through the ComRS signaling system in CDM. We therefore examined the stability of SigX and the effects of inactivated MecA, ClpC or ClpP on the cellular levels of SigX by Western blot analysis of strain XT-His1 (wt) and three mutant background strains, XT-His2 (Δ*mecA*), XT-His3 (Δ*clpC*) and GF-His1 (Δc*lpP*) grown in CDM in response to XIP. The results showed that XIP induced a rapid synthesis of SigX in strain XT-His1 (wt) grown in CDM (Figure 3A). Surprisingly, an increasing level of SigX was detected in this strain throughout the experiment, although relatively stable levels of the protein samples were loaded on the gel, as indicated by the protein loading controls. The results suggested a cellular accumulation of the SigX after its synthesis in CDM. Consistent with these levels of SigX, a prolonged competent state was observed in *S. mutans* UA159 grown under the same condition (Figure 3B). We then examined the effects of deletion of *mecA* (Δ*mecA*), *clpC* (Δ*clpC*) or *clpP* (Δc*lpP*) on the cellular levels of SigX in CDM. The results clearly showed the cellular accumulation of SigX in all three mutant strains grown in CDM (Figure 3C). As indicated by the relative integrated density values (RIDVs) (Figure 3D), the cellular levels of SigX in these mutant strains remained high or even higher than the wild type control strain throughout the experiments. In agreement with such stable levels of SigX, all the strains showed a prolonged competence state for taking up transforming DNA in CDM (Figure 3B). The results clearly show that SigX is relatively stable after the synthesis during competence induction by XIP in CDM, resulting in a prolonged competent state for taking up exogenous DNA.

**Figure 4:**
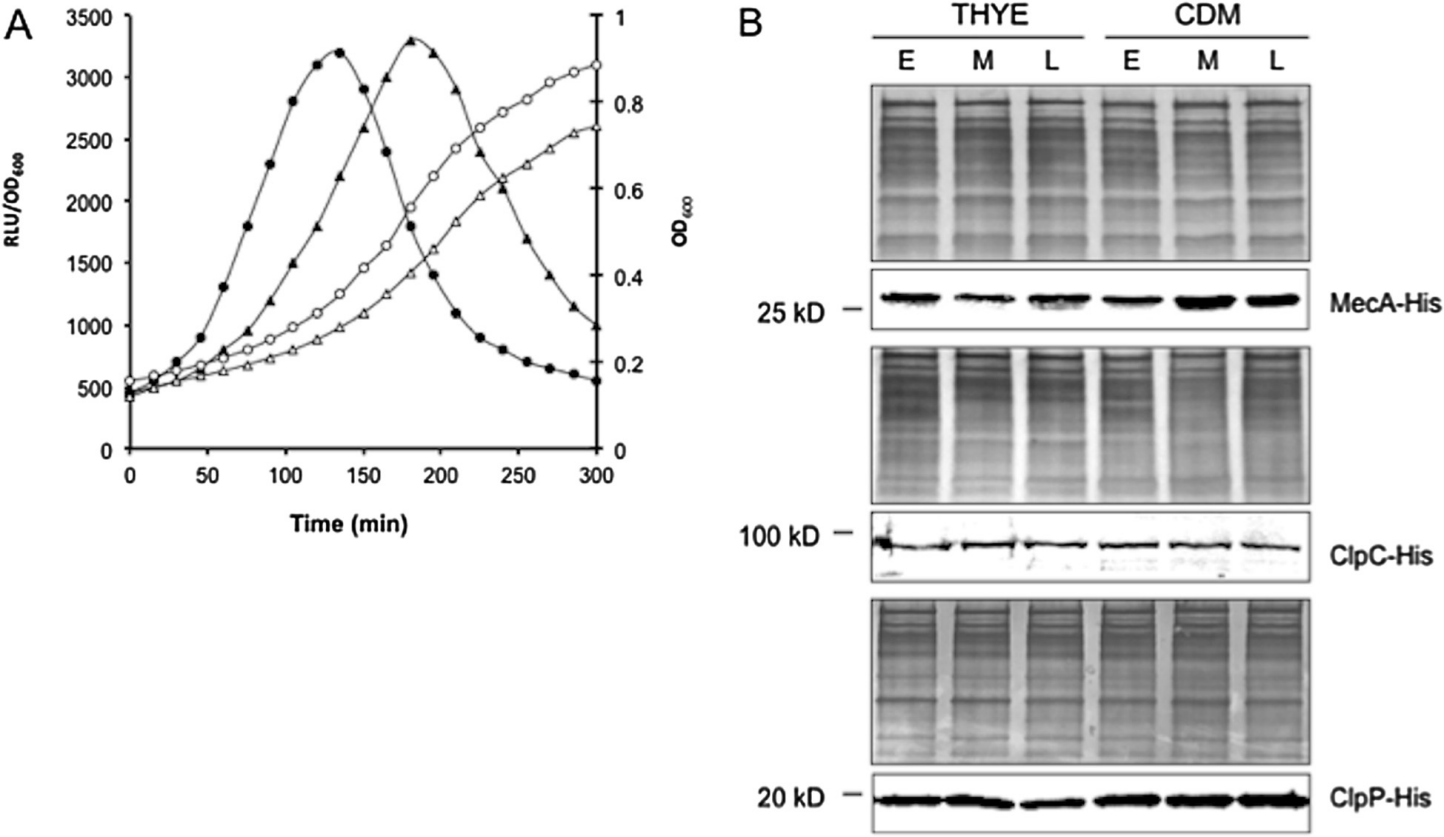
**Effects of the growth conditions on expression of *comX *and cellular levels of MecA, ClpC and ClpP. A**. Luciferase reporter activities (black circles and triangles) of the competence-specific promoter, P*comX,* in *S. mutans* strain XT-Lx1 grown in THYE (open circles) or in CDM (open triangles) in response to CSP or XIP. **B**. Western blot analysis of cellular levels of MecA, ClpC or ClpP in *S. mutans* strains GF-His2 (MecA-His), GF-His3 (ClpC-His) and GF-His4 (ClpP-His) grown in THYE (+CSP) and in CDM (+XIP). The samples were taken from the cultures of these strains during the early- (E), mid- (M) and late- (L) exponential phases to prepare the crude cell lysates. The MecA, ClpC and ClpP were detected by Western blotting using the anti-His antibody, while the protein loading controls were detected using the anti-*S. mutans* antibody.

These findings led to two hypotheses: [1] that the stable levels of SigX in CDM might be due to a persistent expression of *comX*, which overcome degradation of SigX, or [2] that the growth conditions in CDM might negatively affect expression of MecA, ClpC or ClpP, resulting in insufficient levels of one or more of these proteins for SigX degradation. To test the first hypothesis, we examined kinetics of the luciferase reporter activities of the competence-specific promoter, P*comX*, in *S. mutans* wild type background strain XT-Lx1 grown in CDM or in THYE in response to XIP or CSP. We found that the specific luciferase activities of the *comX* promoter, P*comX*, still showed transient increases in response to XIP and then declined afterward, giving a pattern similar to that in THYE (Figure 4A). However, the luciferase reporter activities of this strain in CDM peaked around 200 min post XIP, which was about 1.5 hour later than the peak level in THYE post CSP. The pattern in the luciferase reporter activities appeared to be a reflection of the slower growth rate of this strain in CDM, although the reporter activities in CDM declined slightly later than those in THYE. We then began to test the second hypothesis by examining cellular levels of MecA, ClpC and ClpP in *S. mutans* strains, GF-His2 (MecA-His), GF-His3 (ClpC-His) and GF-His4 (ClpP-His), grown in both THYE (+CSP) and CDM (+XIP) by Western blot analysis. The results showed that considerable levels of MecA, ClpC and ClpP were detected during the (early, mid and late) exponential growth phases in both THYE and CDM (Figure 4B). No significant difference was observed in the cellular levels of these proteins between THYE and CDM. The results suggest that the stable levels of SigX detected in CDM may not necessarily result from variations in the cellular levels of MecA, ClpC or ClpP, but rather from an unknown mechanism that may negatively affect regulated proteolysis of SigX when *S. mutans* is grown in CDM.

### MecA mediates the formation of a ternary SigX-MecA-ClpC complex

**Figure 5:**
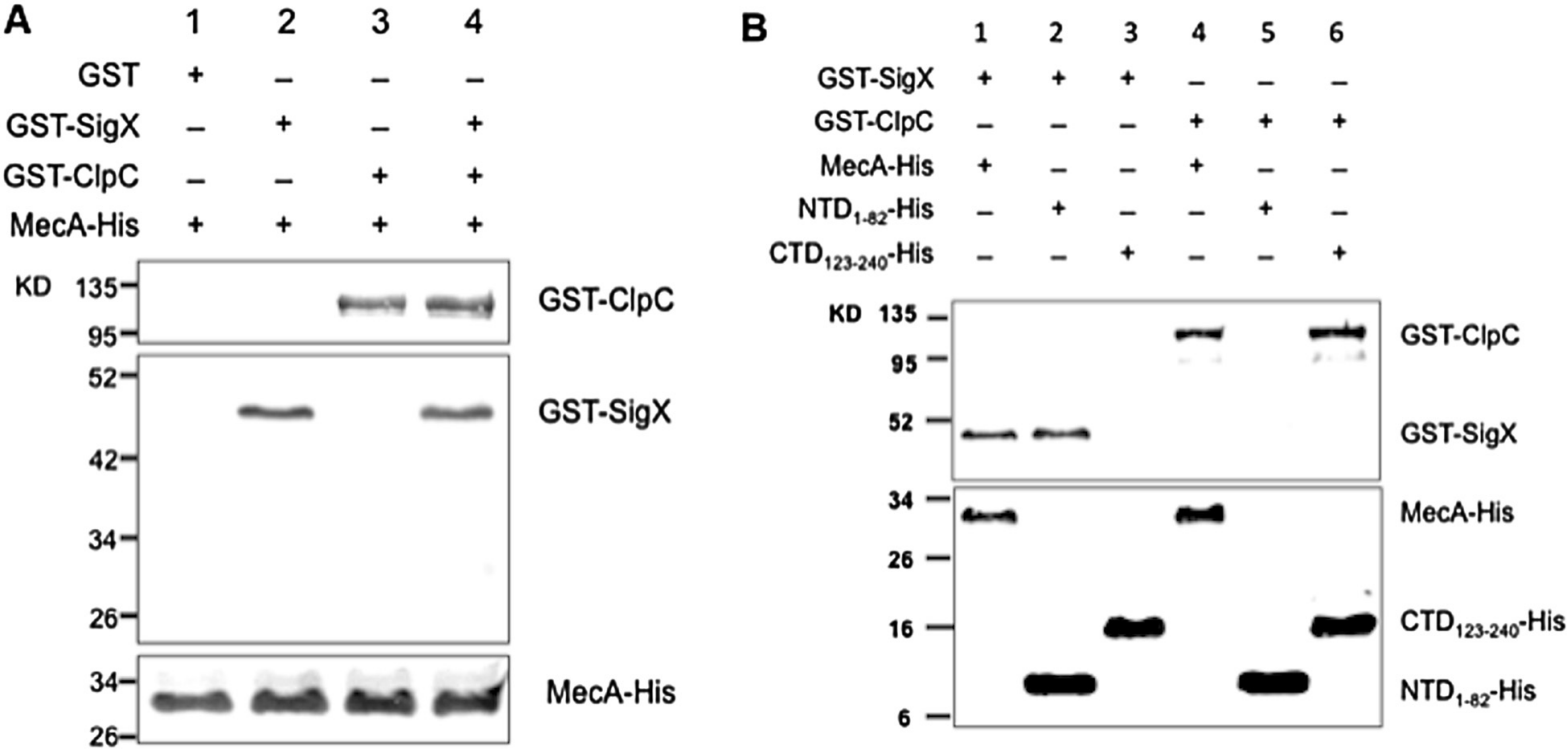
***In vitro *protein-protein interactions of MecA, SigX and ClpC by co-elution experiments. A**. Protein-protein interactions were initiated by incubating the protein mixtures as follows: (1) MecA-His and GST (control), (2) MecA-His and GST-SigX, (3) MecA-His and GST-ClpC and (4) all three proteins. The reactions were incubated at 37°C for 1 hour before applied to Ni-charged resin for co-elution. The elution samples were then analyzed by Western blotting using the anti-His or anti-GST antibody, respectively. **B**. Effects of deletion of the *N*-terminal domain or *C*-terminal domain of MecA on its interaction with SigX or ClpC. Lane 1 and 4 indicate the interactions of the full-length MecA with both SigX and ClpC. Lane 2 indicates an interaction of the *N*-terminal domain (NTD_1–82_) with SigX, but not with ClpC (Lane 5). Lane 6 indicates an interaction of the *C*-terminal domain (CTD_123–240_) with ClpC but not with SigX (Lane 3).

Recent study using bacterial two-hybrid (B2H) system shows that MecA interacts with SigX and ClpC, mediating the formation of a ternary complex [34]. To obtain direct evidence of the interaction among these proteins, we cloned, expressed and produced recombinant proteins of SigX, MecA, ClpC and ClpP using protein expression technology. Each of the resulting proteins carried a His-tag or GST-tag that facilitated subsequent purification and identification of these proteins. SDS-PAGE analysis of these proteins showed successful production of sufficient amounts of all fusion proteins. Their identities and predicted molecular sizes following purification are described in Additional file 1: Figure S1, Additional file 1: Figure S2 and Additional file 1: Figure S3. The fusion proteins were further confirmed by Western blotting using the anti-His or anti-GST antibody. With these purified proteins, we next examined MecA-mediated protein-protein interactions by co-elution experiments using Ni-charged resin. After extensive washings, the proteins were eluted in a buffer and the elution samples were analyzed by Western blotting. As shown in Figure 5A, MecA did not interact with GST-tag (lane 1), but interacted with SigX (lane 2) or ClpC (lane 3) or both (lane 4). The results clearly indicate that MecA protein interacts simultaneously with both SigX and ClpC, likely mediating the formation of a ternary SigX-MecA-ClpC complex.

Since the *N*-terminal and *C*-terminal domains of MecA recognize different protein partners in *B. subtilis*[33-35], we produced two MecA deletion variants from *S. mutans*, the *N*-terminal domain (NTD_1–82_) that had a *C*-terminal deletion, and the *C*-terminal domain (CTD_123–240_) that had a *N*-terminal deletion, based on the sequence alignment of MecA proteins from *B. subtilis* and *S. mutans* strains (Additional file 1: Figure S4). We then examined the effects of a deletion of the *N*-terminal or *C*-terminal domain of MecA on its interactions with SigX and ClpC in *S. mutans*. The results showed that the NTD_1–82_ interacted with SigX (Figure 5B, lane 2), but not with ClpC (Figure 5B, lane 5), suggesting that MecA bound to SigX through the *N*-terminal domain. In contrast, the CTD_123–240_ of MecA did not interact with SigX (Figure 5B, lane 3), but interacted with ClpC (Figure 5B, lane 6), suggesting that MecA bound to ClpC through the *C*-terminal domain. The full-length of MecA interacted with both SigX (Figure 5B, lane 1) and ClpC (Figure 5B, lane 4). Together, the results suggest that MecA interacts with both SigX and ClpC via its *N*-terminal domain binding to SigX and via its *C*-terminal domain binding to ClpC, meditating the formation of a ternary SigX-MecA-ClpC complex.

### MecA mediates an ATP-dependent degradation of SigX by the ClpC/ClpP

**Figure 6:**
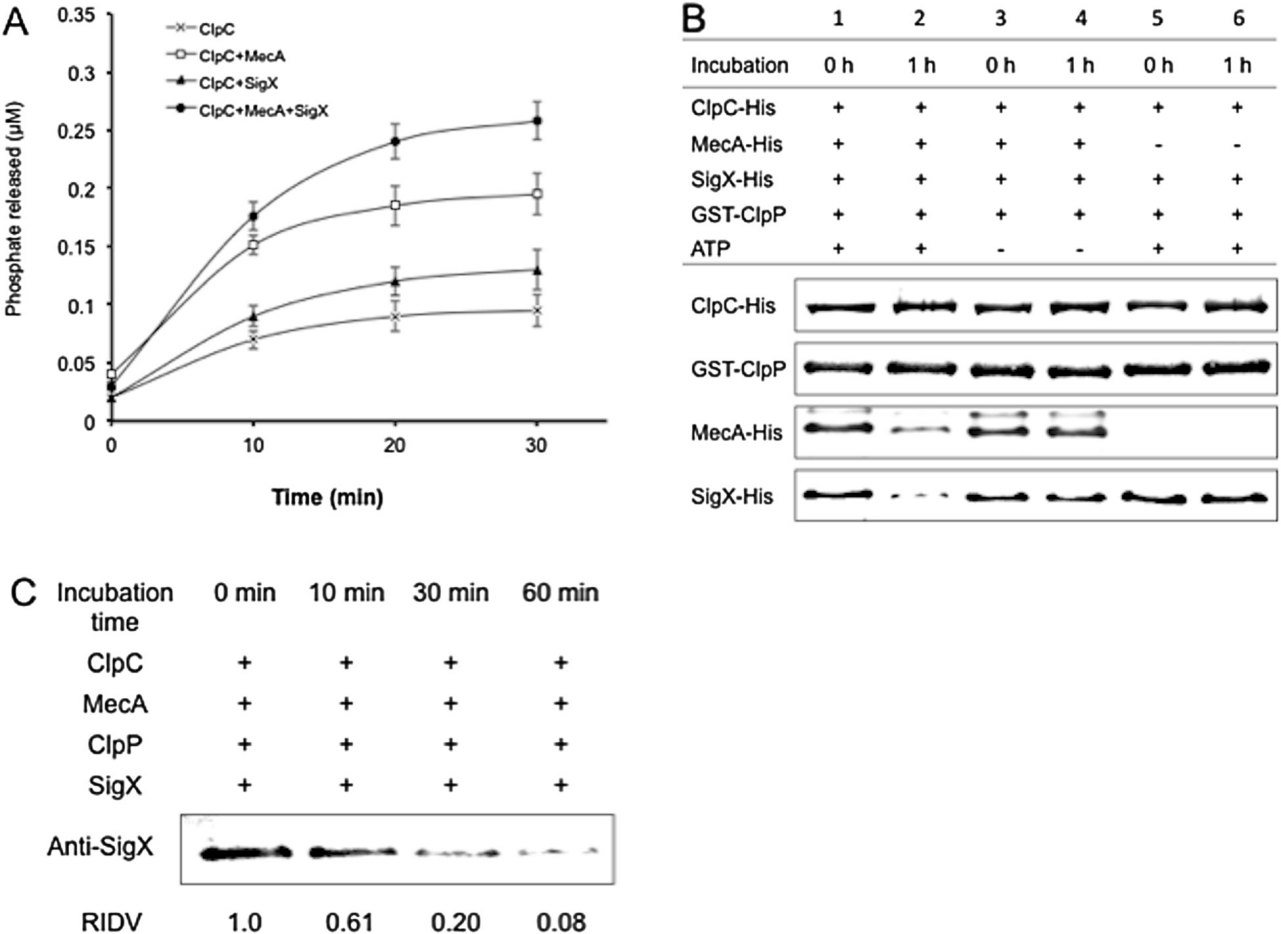
***In vitro *degradation assays to determine MecA-mediated proteolysis of SigX. A**. A colorimetrical assay of ATPase activity of ClpC. The reactions were initiated in a buffer by adding the following protein(s): (1) ClpC alone (black stars), (2) ClpC and MecA (open squires), (3) ClpC and SigX (black triangles), and (4) ClpC, MecA and SigX (black circles). These proteins were treated with PreSciession protease to remove GST-tag before used for the assay. **B**. The degradation reactions were initiated in a reaction buffer, including Group 1: ClpC-His, MecA-His, SigX-His, GST-ClpP and ATP (lane 1 and 2), Group 2: ClpC-His, MecA-His, SigX-His and GST-ClpP without addition of ATP, and Group 3: ClpC-His, SigX-His, GST-ClpP and ATP without MecA-His (lane 5 and 6). Aliquots of samples were taken from the reactions to assess degradation results by Western blot analysis of the interacting proteins using the anti-His or anti-GST antibody. **C**. Degradation assay of the reaction mixture containing ClpC, MecA, SigX and ClpP after removal of the GST-tag by PreScission protease cleavage. The SigX was detected by Western blotting using the anti-SigX antibody and the remaining SigX protein on the membrane was scanned and converted as RIDV values.

We next examined whether MecA targeted SigX for degradation by the protease complex ClpC/ClpP. We first determined the ATPase activity of the ClpC protease by an ATPase activity assay [31,37]. The results showed that ClpC alone exhibited a very low ATPase activity, but the presence of MecA increased the ATPase activity of ClpC (Figure 6A). Interestingly, adding both MecA and SigX into the reaction increased the ATPase activity of ClpC by nearly 2-folds, although MecA or SigX alone exhibited little ATPases activity (data not shown). The results confirmed that the ATPase activity of ClpC depended on the presence of MecA and ATP, and the ATPase activity could be further enhanced by the presence of SigX. We then examined MecA-mediated proteolysis of SigX using a degradation assay. The reactions were mixed in the ATPase assay buffer by adding ClpC, MecA, SigX, ClpP and ATP. Aliquots of the samples were taken to assess degradation results by Western blot analysis of the interacting proteins. The results showed that degradation of SigX occurred in the presence of MecA, ClpC, ClpP and ATP in one-hour incubation (Figure 6B, lane 2). No detectable degradation of SigX was observed without MecA (lanes 5–6) or ATP (lane 3–4), suggesting that degradation of SigX required the presence of MecA and ATP. Interestingly, a similar level of degradation of MecA was also observed in the reaction (Figure 6B, lane 2), suggesting that MecA might serve as a degradation tag. To confirm the results, we also incubated these proteins for degradation after GST-tag was removed with PreScission protease. We then assessed degradation of SigX by Western blotting using the anti-SigX antibody. A similar result was observed, which showed that SigX degradation occurred as quickly as around 10 min (Figure 6C). Approximately 80% of SigX protein was degraded after 30-min incubation and over 90% of SigX was degraded after 60-min incubation, as indicated by the RIDV values of the remaining SigX in the reaction. In addition, we found that neither NTD_1–82_ nor CTD_123–240_ mediated degradation of SigX under the same condition (data not shown).

## Discussion

In the Genus *Streptococcus*, the alternative sigma factor, SigX encoded by *comX,* is the competence-specific master regulator that is transiently induced to drive expression of numerous late competence genes essential for DNA uptake and recombination [14-16]. The activity and stability of SigX during competence development have been relatively well studied in the model organism *S. pneumoniae,* which involves a complex regulatory network and multiple components such as ComW, DprA and ClpE/ClpP [28-30,38,39]. However, it was unclear whether the same mechanisms might be used for regulation of the activity and stability of SigX in other members of streptococci that primarily used a ComRS signaling system to control transcription of *comX* and competence. In this study, we provide evidence that *S. mutans* regulates the cellular level and stability of SigX by a different mechanism that involves MecA-mediated proteolysis of SigX by the protease ClpC/ClpP. We have found that adaptor protein MecA in *S. mutans* targets SigX for degradation by the ClpC/ClpP when this organism is grown in a complex medium, a growth condition that is sub-permissive for competence development [25-27]. Under this growth condition, the synthesis of SigX is transiently induced by CSP, but the SigX is quickly degraded during the escape from competence. The degradation of SigX by the protease ClpC/ClpP requires the presence of MecA, which plays an important role in the recognition and targeting of SigX under this growth condition. By analysis of *in vitro* protein-protein interactions, we demonstrate that MecA interacts with both SigX and ClpC and mediates the formation of a ternary SigX-MecA-ClpC complex, which triggers the ATP-dependent degradation of SigX in the presence of ClpP. The result confirms previous findings of SigX-MecA-ClpC interactions using a bacterial two-hybrid system [34]. We also demonstrate that MecA binds to SigX via its *N*-terminal domain (NTD_1–82_) and to ClpC through its *C*-terminal domain (CTD_123–240_). A deletion of the NTD or the CTD of MecA abolishes its binding to SigX or ClpC, failing to mediate the formation of the ternary complex and regulated proteolysis of SigX. These results suggest that the NTD of MecA is essential for substrate recognition, while the CTD is required for the assembly of the protease complex ClpC/ClpP and stimulates the ATPase activity of ClpC. The evidence support the notion that MecA plays an important role in regulated proteolysis of SigX by its substrate recognition and regulation of the assembly and proteolytic activity of the ATP-dependent ClpC/ClpP protease.

This notion is highly consistent with the recent report by Wahl *et al.*[40], who have demonstrated by *in vitro* experiments that MecA in *S. thermophilus* also targets SigX for degradation by ClpCP. The formation of MecA-ClpCP proteolytic complex is suggested to be an additional locking device to regulate the stability of SigX and competence in *S. thermophilus* under inappropriate conditions. In particular, these researchers have identified an 18-residue sequence (predicted loop) in the *N*-terminal domain of SigX as a key determinant for the interaction with MecA and subsequent SigX degradation. Interestingly, both *S. mutans* and *S. thermophilus* have 7 out of 10 highly conserved residues, while *S. pneumoniae* only shares 3 similar residues with the loop of *S. thermophilus*[40]. This suggests that SigX from *S. pneumoniae* has diverged during evolution in such a way that this species lacks the recognition of SigX by MecA protein. This divergence explains why an inactivation of MecA in *S. pneumoniae* has no major impact on *in vivo* degradation of SigX but dramatically stabilizes ComW [28,29].

Another important finding is that changes in the expression of MecA dramatically affect its regulated proteolysis of SigX. We demonstrate that a deletion of *mecA* allowed the cellular accumulation of SigX, resulting in a prolonged competence state (X state). However, the deletion of MecA is insufficient to activate competence under this growth condition, since the ComRS but not MecA is required for transcriptional activation of *comX* and genetic competence [18,34]. In contrast, an overexpression of MecA increases regulated proteolysis of SigX, accelerating the escape of the cells from competence. The results strongly suggest that MecA in *S. mutans* actually acts as an anti-sigma factor in the regulation of cellular levels of SigX. The implication of an adaptor protein that acts as an anti-sigma factor in Clp protease-dependent manner was previously reported in both Gram-negative and Gram-positive bacteria [3-8]. In the case of *E. coli*, for example, adaptor protein RseB specifically targets Sigma S (RpoS) to ClpX/P for degradation, regulating the levels of RpoS in the stationary phase or general stress response in *E. coli*[11,41]. Another example is adaptor protein RsiW that acts as an anti-sigma factor in the control of sigma factor W (σ^W^) during pH and salt stresses in *B. subtilis*[42]. In addition, *B. subtilis* regulates the activity of an extracytoplasmic function (ECF) sigma factor, σ^V^, to cope with the cell envelope stress induced by antibiotics. The σ^V^ activity is also regulated by proteolysis of an anti-sigma factor RsiV [43]. Thus, regulated proteolysis of a master regulator by adaptor protein that acts as an anti-sigma factor in regulation of development and stress response is well conserved among bacteria, including streptococci [41-45]. This reinforces the importance of fine control of competence and stress response that have been positively selected through microbial evolution. Currently, little is known of how MecA is regulated in streptococci. This is important, since MecA is ubiquitous among streptococci and its homologs are found in the complete genomes of all species sequenced so far [34,35]. In a most recent study, Wahl *et al.* have provided convincing *in vitro* evidence that confirms the importance of MecA-ClpCP complex in the posttranslational regulation of SigX in *S. thermophilus*[40]. However, their work has not described how MecA is regulated *in vivo* under test growth conditions. In our study, we have explored this question by some *in vivo* experiments, but limited evidence is insufficient to draw any conclusion. Further study is clearly needed.

Interestingly, MecA-regulated proteolysis of SigX appears to be ineffective when *S. mutans* is grown in CDM, a growth medium that is permissive for competence development [25-27]. In this medium, the synthesis of SigX is initially induced by XIP, but a high level of SigX can persist throughout the experiment, suggesting a cellular accumulation of SigX following the initial synthesis. Consistent with such a stable level of the SigX, *S. mutans* shows a prolonged competence state for taking up transforming DNA in CDM. These results are surprising, because MecA in the wild type *S. mutans* strain can effectively mediate degradation of SigX by the protease ClpC/ClpP after initial synthesis of SigX in THYE. The results suggest that the cellular accumulation of SigX in CDM may result from one or two possible changes: an overexpression of SigX in CDM or ineffective degradation of SigX in CDM or both. Recent studies show that *S. mutans* can develop a high level of competence for genetic transformation when grown in CDM in response to XIP [18,25-27]. The competence state induced by XIP in CDM can persist for hours, suggesting an increased or persistent expression of *comX* during competence induction through the ComRS pathway in CDM. To test this model, we examined kinetics of the luciferase reporter activities of the competence-specific promoter, P*comX*, in response to XIP in CDM, in comparison with the reporter activities of the same strain grown in THYE in response to CSP. The data from this work do not fully support this suggestion, since the luciferase reporter activities in CDM still show a transient increase with a peak level and then decline, a pattern similar to that in THYE. This indicates that expression of *comX* through ComRS system is still a transient event at transcriptional level, although the reporter activities in CDM appear to last longer than those in THYE. We interpret this might be partially because the cells grew slower in CDM than that in THYE. Thus, the evidence from the luciferase reporter assays appears to be insufficient to explain such high levels of cellular accumulation of SigX in CDM. This raises a question why such high levels of SigX cannot be effectively degraded by the protease ClpC/ClpP if this proteolytic system works well in CDM. This has led us to hypothesize that the growth condition in CDM might negatively affect expression of MecA, ClpC or ClpP, resulting in insufficient levels of one or more of these proteins required for SigX degradation. We therefore compared the cellular levels of MecA, ClpC and ClpP in both CDM and THYE media. Surprisingly, we detected relatively stable levels of MecA, ClpC and ClpP from the exponentially growing cultures of all the strains. No significant difference was observed in the cellular levels of each of these proteins between THYE and CDM. The data suggest that the stable levels of SigX in CDM may not necessarily result from variations in the cellular levels of MecA, ClpC or ClpP, but rather from an unknown mechanism that may negatively affect regulated proteolysis of SigX in CDM.

In *B. subtilis*, ComK is the master transcriptional regulator required for competence activation [31,32]. Under non-competence conditions, ComK is sequestered in a MecA and ClpC complex, which triggers degradation of ComK in the presence of ClpP [31-33,37,44]. For competence to develop, degradation of ComK must be blocked by a small protein ComS, which acts as an anti-adaptor to bind to MecA and displace the master regulator ComK [31-33]. Binding of ComS to MecA protects ComK from degradation by the ClpC/ClpP and turns on the switch leading to competence activation [32,37]. This system obviously has parallels to the regulation of RpoS degradation, a situation that the substrate is degraded, dependent upon an adaptor protein, and regulation of degradation is achieved by regulated synthesis of an anti-adaptor protein [11,41]. It is currently unknown whether the *S. mutans* genome contains a gene encoding such an anti-adaptor to regulate MecA-mediated proteolysis of SigX. However, the evidence from this study suggests the possibility that such an anti-adaptor protein likely exists, since SigX is relatively well protected from degradation in CDM, and since little difference is detected in the cellular levels of MecA, ClpC and ClpP from the exponential growth cultures in CDM and THYE. We speculate that the growth condition in CDM may affect the synthesis or the activity of such an anti-adaptor, which in turn affects MecA-mediated proteolysis of SigX. However, we do not completely rule out the possibilities that other factors may be involved in the regulation of the activity of the protease complex ClpC/ClpP in CDM [45,46] and that both overexpressed SigX and ineffective proteolysis of SigX mediated by MecA are responsible for the cellular accumulation of SigX under this growth condition. Further study may be needed to investigate these questions.

## Conclusions

In summary, we provide evidence that adaptor protein MecA in *S. mutans* plays a central role in recognizing and targeting SigX for degradation by the protease ClpC/ClpP. Thus, MecA actually acts as an anti-sigma factor to regulate the stability of SigX during competence development. Our findings are highly consistent with a recent report that shows a similar mechanism involved in the regulation of the stability of SigX and genetic competence in *S. thermophilus*[40]. We have also found that changes in expression of MecA may profoundly affect the stability of SigX, although further study is needed to elucidate *mecA* regulation. Interestingly, MecA-regulated proteolysis of SigX appears to be ineffective when *S. mutans* is grown in CDM medium, suggesting the possibility that an unknown mechanism may be involved in negative regulation of MecA-mediated proteolysis under this condition. We speculate that MecA-regulated proteolysis may finely control the cellular levels of SigX to prevent the entry into competence under inappropriate conditions or to secure the timely escape of the cells from competence when *S. mutans* encounters a stressful condition.

## Methods

### Bacterial strains, media and growth conditions

Bacterial strains and plasmids used in this study are listed in Table 1. *S. mutans* wild-type strain UA159 was grown on Todd-Hewitt medium plus 0.3% yeast extract (THYE), whereas all constructed strains derived from *S. mutans* UA159 were maintained on THYE plus appropriate antibiotic(s). For some experiments, *S. mutans* strains were grown in a chemically defined medium (CDM), which was made in the detail as described previously [25,34]. *Escherichia coli* host strains for molecular cloning were grown in Luria-Bertani (LB) medium supplemented with an appropriate antibiotic(s).

**Table 1:** Bacterial strains and plasmids used in this study

**Strains**	**Relevant characteristics**	**Source or reference**
***S. mutans***		
UA159	Wild type, the genome sequence reference strain	48
XT-D1	UA159 ∆*comX*::*spec*, Spec^r^	34
XT-D3	UA159 ∆*mecA::erm*, Erm^r^	34
XT-D4	UA159 ∆*mecA::spec*, Spec^r^	34
XT-D7	UA159 ∆*clpC::kan*, Kan^r^	This study
XT-D8	UA159 ∆*clpP::kan*, Kan^r^	This study
XT-Lx1	UA159 carrying pWAR304*,* Em^r^*,*	34
XT-His1	UA159 carrying pSigX-His, Spec^r^	34
XT-His2	XT-D3 carrying pSigX-His, Em^r^*,* Spec^r^	34
XT-His3	XT-D7 carrying pSigX-His, Kan^r^*,* Spec^r^	34
GF-His1	XT-D8 carrying pSigX-His, Kan^r^*,* Spec^r^	This study
GF-His2	UA159 carrying pMecA-His, Spec^r^	This study
GF-His3	UA159 carrying pClpC-His, Spec^r^	This study
GF-His4	UA159 carrying pClpP-His, Spec^r^	This study
GF-Ox	UA159 pDL277::Pldh-mecA-His, Spec^r^	This study
***E. coli***		
DH5α	Cloning host	Invitrogen
XL1-blue	Cloning host	Stratagene
BL21(DE3)	Recombinant protein expression strain	Novagen
BL21(DE3)pLysS	Recombinant protein expression strain	Novagen
**Plasmids**		
pGEX-6P-1	*E. coli* expression vector (GST-tag), Amp^r^	GE
pGF-SigX	pGEX-6P-1::*comX*, GST-tag fusion, Amp^r^	This study
pGF-MecA	pGEX-6P-1::*mecA*, GST-tag fusion, Amp^r^	This study
pGF-ClpC	pGEX-6P-1::*clpC*, GST-tag fusion, Amp^r^	This study
pGF-ClpP	pGEX-6P-1::*clpP*, GST-tag fusion, Amp^r^	This study
pET-20b(+)	*E. coli* expression vector (6His-tag), Amp^r^	Novagen
pGF2-SigX	pET-20b::*comX,* His-tag fusion, Amp^r^	This study
pGF2-MecA	pET-20b::*mecA*; His-tag fusion, Amp^r^;	This study
pGF2-ClpC	pET-20b::*clpC*; His-tag fusion, Amp^r^	This study
pGF2-ClpP	pET-20b::*clpP*; His-tag fusion, Amp^r^	This study
pGF2-NTD	pET-20b::*mecA*_**(aa1–82)**_, His-tag fusion, Amp^r^;	This study
pGF2-CTD	pET-20b::*mecA*_**(aa123–240)**_, His-tag fusion, Amp^r^;	This study
pGF3-P + X	pET-20b::Promoter + *comX,* His-tag fusion, Amp^r^	This study
pDL277	*E. coli-Streptococcus* shuttle vector, Spec^r^	47
pSigX-His	pDL277::Promoter + *comX*-His, Spec^r^	34
pGF3-PA	pET-20b::Promoter + *mecA,* His-tag fusion, Amp^r^	This study
pMecA-His	pDL277::Promoter + *mecA*-His, Spec^r^	This study
pGF3-PC	pET-20b::Promoter + *clpC,* His-tag fusion, Amp^r^	This study
pClpC-His	pDL277::Promoter + *clpC*-His, Spec^r^	This study
pGF3-PP	pET-20b::Promoter + *clpP,* His-tag fusion, Amp^r^	This study
pClpP-His	pDL277::Promoter + *clpP*-His, Spec^r^	This study
pXT-Pldh	pDL277::Promoter of *ldh* (Pldh), Spec^r^	This study
pMecA-Ox	pXT-Pldh::MecA-His, Spec^r^	This study
pWAR303	A shuttle vector containing promoterless *luxAB,* Em^r^	18
pWAR304	pWAR303 having a fusion of P*comX::luxAB*, Em^r^	18

### Assay for competence activation

A modified competence assay was carried out to monitor competence activation of *S. mutans* strains in response to CSP or XIP [18,34]. *S. mutans* UA159 was used as a positive control, while a *comX* deletion mutant (Δ*comX*) was used as a negative control. All the strains were grown either in THYE medium with use of CSP (SGSLSTFFRLFNRSFTQA) or in CDM with use of XIP (GLDWWSL). Both peptides were commercially synthesized with 90% purity (BioBasic Inc., Ontario). Each peptide was freshly dissolved in sterile distilled water as 1.0 mM, which was further diluted to a final concentration of 500 nM during the experiments. In THYE, an aliquot of CSP was added into the cultures that reached to the early mid-log phase (O.D_600_ ≈ 0.3). In CDM, an aliquot of XIP was added into the cultures that reached the mid-log phase (O.D_600_ ≈ 0.45). To study kinetics of transformation, aliquots (0.5 ml) of samples were withdrawn in duplicate at 15-min intervals and exposed to a transforming DNA (either 1 μg/ml of plasmid DNA or 10 μg/ml of the chromosomal DNA conferring an antibiotic marker) for 30 min. Each samples was added with 10 ng/ml DNases and continued incubation for 60 min. The cell suspensions were taken from each culture, serially diluted and spread on THYE plates with an antibiotic for selection of positive transformants or on THYE plates for total viable cell counts. Transformation frequency was expressed as the ratio of the number of transformants (antibiotic-resistant colonies) to the total number of recipient cells per milliliter of cell suspension.

### Protein extraction and Western blot analysis of the cellular SigX

To examine cellular levels of SigX in *S. mutans*, we used a standard protocol to induce competence and collected samples to prepare the crude protein extracts for Western blot analysis of SigX. The *S. mutans* strains that were constructed to carry a shuttle vector pSigX-His were grown in THYE or CDM to induce competence by either CSP or XIP. Aliquots (4 ml) of samples were taken at different times to prepare crude protein lysates after adjustment of cell densities (OD_600_) to 0.3 in THYE or to 0.5 in CDM by dilution. The cellular levels of SigX in *S. mutans* strains were determined by Western blot analysis. All crude protein samples were resolved on SDS-PAGE gels using a Bio-Rad Mini-Protein II gel apparatus (BioRad). The proteins on the gels were transferred to polyvinylidene difluoride (PVDF) membrane that was then cut into two: one for Western blotting using an anti-His antibody (GeneScript, NJ) and another used as a total protein loading control detected by Western blotting using an anti-*S. mutans* (serotype C) antibody (Abcam, MA). The membranes were blocked with 5% fat-free milk in TBS-T buffer at 4°C for overnight and added with a primary antibody (1:3,000 dilution) and incubated for 90 min. After three washes, the membranes were added with the secondary antibody (1:10,000 diluted anti-rabbit IgG conjugated to alkaline phosphatase or AP). The membranes were incubated for one hour, washed and detected with the AP detection reagents (Novagen). The protein profiles were then examined and photographed using FluorChem SP image system (Alpha Innotech). To quantify the intensities, all protein bands were scanned using UN-SCAN-IT software 6.1 (Silk Sci. Inc.) and the data were converted as relative integrated density values (RIDV), which were normalized to a maximum 1.0 for each strain [16,29].

### Luciferase activity assay

To monitor the activity of the competence-specific promoter, P*comX,* in response to CSP or XIP, luciferase reporter activity was assayed using a *luxAB* transcriptional reporter strain, *S. mutans* XT-Lx1 [34]. The bacterial growth and the luciferase reporter activity were assayed at regular intervals in a microtiter plate reader (Synergy HT, BioTek, USA), which read both bioluminescence and optical density at 600 nm. One percent of nonanal (Sigma-Aldrich) was used for the assay, since LuxAB-catalyzed luciferase activity requires the presence of nonanal as a substrate. Briefly, aliquots (300 μl) of cell cultures were transferred to the wells of a pre-warmed (37°C) microtiter plate. Aliquots (50 μl) of the solution containing 1% nonanal (diluted in mineral oil) in a volatile form were placed in the spaces between the wells of the microplate, which was then covered with the lip. The microtiter plate was placed in the pre-warmed reader for incubation and readings at every 15-min interval for continuous 5 hours. The results were expressed in relative luminescent units (RLU) divided by cell density of the cultures.

### Construction of plasmids pMecA-His, pClpC-His and pClpP-His

To examine the effects of growth conditions on expression of MecA, ClpC and ClpP, we constructed three plasmids, pMecA-His, pClpC-His and pClpP-His, by a two-step cloning strategy [34]. In the first step, we amplified an entire target gene (except stop codon) from the *S. mutans* genome using primers with two restriction sites, EcoR1 or BamH1 and Notl(-B1) (Table 2). The PCR fragments were cloned into an expression vector pET-20b(+) (Novagen), generating plasmids pGF3-PA, pGF3-PC and pGF3-PP, respectively. In the second step, we used a new backward primer with a restriction site BamH1(-B2) or Not1(-B2) and the forward primer to amplify each insertion and its *C*-terminal His-tag from these plasmids and cloned into an *E. coli-Streptococcus* shuttle vector pDL277 [47]. The newly constructed plasmids were confirmed by PCR and restriction analysis, and the confirmed plasmids were designated as pMecA-His, pClpC-His and pClpP-His. We transformed these plasmids into *S. mutans* UA159 to generate three strains, GF-His2, GF-His3 and GF-His4, which allowed detection of His-tagged proteins by Western blotting using the anti-His antibody. We then examined cellular levels of these proteins by growing the strains under tested conditions. Aliquots of cell suspension were taken from the cultures at different times to prepare crude protein lysates after adjustment of the cell density. The cellular levels of His-tagged proteins were analyzed by Western blotting using the anti-His-tag antibody (GeneScript, NJ, USA).

**Table 2:** Primers Used in This Study

Primers*	**Nucleotide Sequence (5′ - > 3′)**	**Purpose**
ComX-F1	CG*GGATCC*ATGGAAGAAGATTTTGAAATTG	GST fusion
ComX-Not1-B	AA*GCGGCCGC*AGGTACAATCACATGTTCCATTC	
MecA-F1	CG*GGATCC*ATGGAAATGAAACAAATCAGC	GST fusion
MecA-Not1-B	AA*GCGGCCGC*AGGTATCATCTAGCTTATCCAATC	
ClpC-F1	CG*GGATCC*CCATCTATGACCGATTACTCA	GST fusion
ClpC-Not1-B	AA*GCGGCCGC*AGGACACTGAAGTAGGGTAAAG	
ClpP-F1	CG*GGATCC*ATGATTCCTGTAGTTATTGA	GST fusion
ClpP-Not1-B	AA*GCGGCCGC*ACATGGTTTTTCTACTCAAC	
ComX-BamH1-F	CG*GGATCC*CATGGAAGAAGATTTTGAAATTG	His fusion
ComX-B3	AA*GCGGCCGC*TTTTTCCTTAAAATCACTTAATTT	
MecA-BamH1-F	CG*GGATCC*CATGGAAATGAAACAAATCAGC	His fusion
MecA-B3	AA*GCGGCCGC*TCCAATCATTTGTAATTCTTGC	
ClpC-BamH1-F	CG*GGATCC*CCCATCTATGACCGATTACTCA	His fusion
ClpC-B3	AA*GCGGCCGC*TACAATATCAAATTTTAATTTTTC	
ClpP-BamH1-F	CG*GGATCC*CATGATTCCTGTAGTTATTGA	His fusion
ClpP-B3	AA*GCGGCCGC*TTTTAATTCATTATTTTCCA	
NTD-BamH1-F	CG*GGATCC*GATGGAAATGAAACAAATCAGC	NTD-His fusion
NTD-Not1-B	AA*GCGGCCGC*TTTATTGATTTCTGACTTAGTGACA	
CTD-BamH1-F	CG*GGATCC*GTTGGCTGAAATTGAAAAAAGAGA	CTD-His fusion
CTD-Not1-B	AA*GCGGCCGC*TCCAATCATTTGTAATTCTTGCAG	
MecA-EcoR1-F1	CG*GAATTC*CACTTTCTTTCTCGGAATACC	MecA-His
MecA-Not1-B1	AA*GCGGCCGC*TCCAATCATTTGTAATTCTTGC	
MecA-BamH1-B2	CG*GGATCC*CAAAAAACCCCTCAAGACC	
ClpC-BamH1-F	CG*GGATCC*TAGCGAGCAAGTTTTTACA	ClpC-His
ClpC-Not1-B1	AA*GCGGCCGC*TATGAAAAATAGACTAGATAAGTAT	
ClpC-Not1-B2	AA*GCGGCCGC*TACAATATCAAATTTTAATTTTTC	
ClpP-EcoR1-F	CG*GAATTC*CGAGCTTAAAATGGGATAAC	ClpP-His
ClpP-Not1-B1	AA*GCGGCCGC*GTATTTTAATTCATTATTTTCCAT	
ClpP-Not1-B2	AA*GCGGCCGC*TTTTAATTCATTATTTTCCATGA	
Pldh-Sac1-F	C*GAGCTC*CCGAGCAACAATAACACTC	Pldh cloning
Pldh-Spe1-B	GG*ACTAGT*AACATCTCCTTATAATTTATTAAGTATATATTCTAT	
MecA-Ox-F	GG*ACTAGT*ATGGAAATGAAACAAATCAGC	MecA over expression
MecA-Ox-B	CG*GGATCC*CAAAAAACCCCTCAAGACC	

*:The engineered restriction sites are italicized: *GGATCC*: BamH1; *GCGGCCGC*: Not1*; GAATCC*: EcoR1; *GAGCTC*: Sac1; *ACTAGT*: Spe1.

### Construction of a *S. mutans* strain that constitutively expresses MecA

To further examine the effect of MecA on the cellular level of SigX, we also constructed a constitutively expressed MecA strain by a two-step cloning strategy. In the first step, we amplified the *mecA*-6His from pMecA-His by PCR using primers mecA-Ox-F and mecA-Ox-B, and also the promoter region of a constitutively expressed gene *ldh* encoding lactate dehydrogenase using primers Pldh-F and Pldh-B from the *S. mutans* UA159 genome [48]. The *ldh* amplicon was digested with Sac1 and Spe1 and the *mecA*-6His amplicon was digested with Spe1 and BamH1. Both fragments were ligated with a shuttle vector pDL277 digested with Sac1 and BamH1. The ligation product was transformed into an *E. coli* host DH5α. The transformants grown on LB agar plates plus spectinomycin (50 μg/ml) were selected for genetic confirmation by PCR and restriction digestion analysis. A confirmed clone, designated as pMecA-Ox, was transformed into *S. mutans* UA159 to generate a new strain, GF-Ox (MecA-Ox), which allowed detection of the constitutively expressed MecA by Western blot analysis using the anti-His antibody. To detect the cellular levels of SigX in the MecA overexpression strain by Western blotting, we had an anti-SigX antibody prepared commercially in rabbits. The antigen design, animal immunizations, antiserum collection and validation were performed with a recommended protocol (Biosynthesis Inc., Lewisville, TX, USA).

### Cloning, expression and production of recombinant proteins

To study *in vitro* protein interactions, we cloned, expressed and produced four recombinant proteins, MecA, SigX, ClpC and ClpP. We also included production of two MecA deletion variants, the *N*-terminal domain (NTD_1–82_) and the *C*-terminal domain (CTD_123–240_), to determine the effects of the *C*-terminal or *N*-terminal deletion of MecA on its interactions with SigX or ClpC. All clones were generated by a standard PCR cloning strategy using specific primers that introduced two restriction sites (Table 2). The resulting PCR products were digested, purified and cloned into both pET-20b(+) expression vector with the C-terminal His-tag (Novagen) and pGEX-6P-1 vector with the N-terminal GST-tag (GE Healthcare), which facilitated subsequent isolation, purification and identification of the fusion proteins. The identities of individual clones were verified by PCR and restriction analysis. Confirmed plasmids were then transformed into *E. coli* BL21(DE3)pLysS cells (Novagen) for over-expression at 30°C by adding 0.5 mM IPTG. The crude proteins were extracted from soluble fraction of cell lysates and further purified using either GST protein purification kit or high affinity Ni-charged resin (GeneScript, NJ, USA) according to the manufacturer’s protocols. All the His- or GST-tagged proteins were verified by Western blot analysis using a monoclonal antibody against the His-tag or GST-tag. All protein concentrations were measured using the standard bicinchoninic acid (BCA) assay (Thermo Scientific). To remove the GST tag from the recombinant proteins, an aliquot (20 U/ml) of PreScission protease (GE healthcare) was added into the GST column bound to GST-tagged proteins and incubated at 4˚C for overnight with gentle shaking. The protease-digested proteins were then eluted with PBS buffer before used for protein analysis.

### *In vitro* protein-protein interaction assays

With the purified recombinant proteins, we next examined MecA-mediated protein-protein interactions by a co-elution experiment with Ni-charged resin (GenScript, NJ) followed by protein detection using the anti-His and anti-GST antibodies. In the first set of the experiment, we determined protein-protein interactions by incubating the protein mixtures as follows: 1) MecA-His (His-tag) and GST-SigX (GST-tag), 2) MecA-His and GST-ClpC, 3) all three proteins, and 4) MecA-His and GST (control). In the second set of the experiment, we determined the effects of MecA deletion variants, NTD_1–82_-His and CTD_123–240_-His, on the protein-protein interactions by incubating protein mixtures as follows: 1) the full-length MecA-His and GST-SigX, 2) NTD_1–82_-His and GST-SigX, 3) CTD_123–240_-His and GST-SigX, 4) the MecA-His and GST-ClpC, 5) NTD_1–82_-His and GST-ClpC and 6) CTD_123–240_-His and GST-ClpC. Each reaction was then mixed with Ni-charged resin and incubated at 4°C with gently shaking for 1 hour. The resin was washed 3X with a buffer (50 mM NaH_2_PO_4_, 300 mM NaCl, 10 mM imidazole, pH 8.0). After extensive washings, the resin was added with 2X SDS sample buffer for elusion. The elution samples were boiled at 95°C for 5 min and centrifuged. The supernatant of the elution samples were analyzed to detect the interacting proteins by Western blotting using the anti-His and anti-GST antibody, respectively.

### ATPase assay

Before degradation assay, the MecA-mediated ATPase activity of ClpC was determined by a colorimetrical assay of released orthophosphate from ATP [37,49]. The assay was initiated at 37°C by adding 0.3 uM ClpC, SigX and MecA in a reaction buffer containing 2 mM ATP, 0.5 mM DTT, 0.1 mM EDTA, 0.5 μg/μl of BSA, 100 mM KCl, 5 mM MgCl_2_ and 25 mM Tris–HCl (pH 8.0). The reaction was incubated for 30 min and stopped by adding a color solution (10.5 mg/ml ammonium molybdate, 0.0034% malachite green, 0.1% Triton X-100) followed by 34% citric acid. The optical density of the samples was read at 650 nm in a microtiter plate reader (Synergy HT, BioTek, USA).

### Degradation assay

To determine whether MecA targeted SigX for degradation by ClpC/ClpP, we performed an *in vitro* degradation assay to examine degradation of SigX protein. The reaction components included SigX (1.0 μM), MecA (1.0 μM), ClpC (2.0 μM), ClpP (2.0 μM) and an ATP regeneration system (10 mM ATP, 0.033 mg/ml of creatine phosphokinase, 1.6 mM creatine phosphate, pH 7.0). A pre-incubation step with ADP (5 mM) was set up to allow the assembly of the proteolytic complex. The reactions were mixed in the ATPase assay buffer by the following order: ClpC, MecA, SigX, ClpP and ATP in an ATP regeneration buffer [31,47]. The reactions were incubated at 30°C for 30 min and removed to 37°C for incubation for an additional hour. Aliquots (50 μl) of samples were taken to assess degradation results by Western blot analysis of the interacting proteins using the anti-His or anti-GST antibody. To confirm the degradation results, we also examined SigX degradation by mixing GST-removed ClpC, MecA, SigX and ClpP with PreScission protease. Aliquots of the samples were taken over times to assess SigX degradation by Western blotting using the anti-SigX antibody. The bands on the membrane were scanned and converted as RIDV values.

### Ethics statement

This research did not involve human subjects, human material, or human data.

## Abbreviations

CDM: Chemically defined medium; CSP: Competence-stimulating peptide; CTD: *C*-terminal domain; NTD: *N*-terminal domain; RIDV: Relative integrated density value; THYE: Todd-Hewitt plus yeast extract; XIP: *sigX*-inducing peptide.

## Competing interests

The authors declare that they have no competing interests.

## Authors’ contributions

GD, XLT and ZAG carried out the experiments. GD designed the primers and performed sequence alignments. YHL designed the project and wrote the manuscript. All authors read and approved the final manuscript.

## Supplementary Material

Additional file 1: Figure S1 Expression of GST-tagged recombinant proteins, GST-SigX, GST-MecA, GST-ClpC and GST-ClpP. **Figure S2.** Expression of His6-tagged recombinant proteins, SigX-His6, MecA-His6, ClpC-His6 and ClpP-His6. **Figure S3.** The identities of eight recombinant proteins purified from the whole-cell lysates of *E. coli* BL21(DE3)pLysS cultures. **Figure S4.** The sequence alignment of MecA proteins from *B. subtilis* 168 (Bs168) and *S. mutans* strains (SmUA159, SmGS-5, SmLJ23 and SmNN2025) were performed using the software of MacVector 9.0 ClusterW. Click here for file
