# Supplementary material for: Regulated proteolysis of the alternative sigma factor SigX in Streptococcus mutans: implication in the escape from competence
Source: BMC Microbiol. 2014 Jul 9;14:183. doi: 10.1186/1471-2180-14-183 (PMC4109385; doi:10.1186/1471-2180-14-183)
Supplement: Additional file 1: Figure S1 — Expression of GST-tagged recombinant proteins, GST-SigX, GST-MecA, GST-ClpC and GST-ClpP. Figure S2. Expression of His6-tagged recombinant proteins, SigX-His6, MecA-His6, ClpC-His6 and ClpP-His6. Figure S3. The identities of eight recombinant proteins purified from the whole-cell lysates of E. coli BL21(DE3)pLysS cultures. Figure S4. The sequence alignment of MecA proteins from B. subtilis 168 (Bs168) and S. mutans strains (SmUA159, SmGS-5, SmLJ23 and SmNN2025) were performed using the software of MacVector 9.0 ClusterW. [file 1471-2180-14-183-S1.pdf]

Regulated Proteolysis of the Alternative Sigma Factor SigX in *Streptococcus mutans*: Implication in the Escape from Competence

Gaofeng Dong[1], Xiao-Lin Tian[1], Zubelda A. Gomez[2] and Yung-Hua Li[1,2]*

[1] Department of Applied Oral Sciences

[2] Department of Microbiology and Immunology

Dalhousie University

Halifax, NS, Canada

Supplement Materials

***: Corresponding Author**

Mailing Address:

5981 University Ave. Rm5215

Halifax, Nova Scotia

Canada, B3H 1W2

Tel: 1-902-494-3063

Fax: 1-902-494-6621

E-mail: yung-hua.li@dal.ca

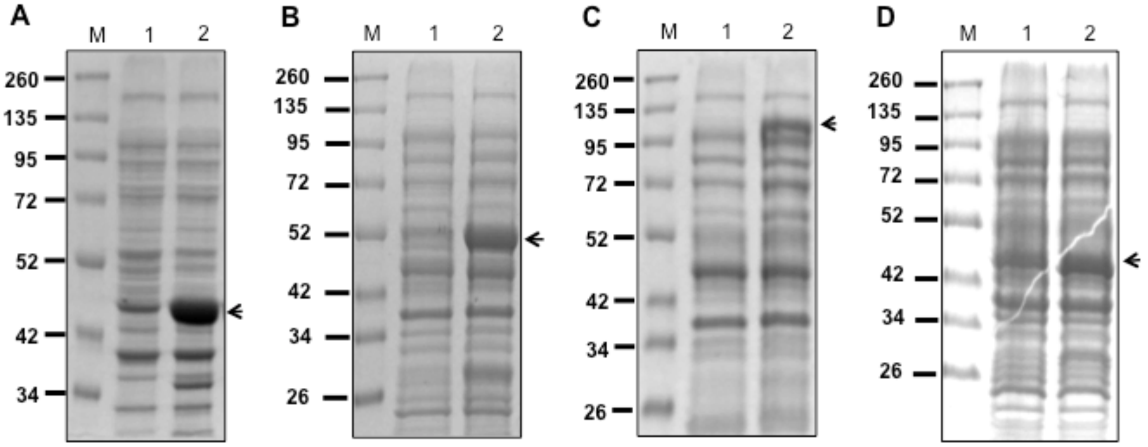

Figure S1. Expression of GST-tagged recombinant proteins. The whole-cell lysates were prepared from the cultures of *E. coli* BL21(DE3)pLysS strains that respectively expressed **A**. GST-SigX, **B.** GST-MecA, **C.** GST-ClpC, and **D.** GST-ClpP. The proteins in the cell lysates were separated in 10% SDS-PAGE gels and stained with Coomassie blue. M: molecular marker in Kilodalton; 1.The cell lysates prepared from the cultures without addition of IPTG; 2: The cell lysates prepared with addition of 0.5 mM IPTG for 2 hours. Arrows indicate expressed GST-tagged proteins with predicted molecular sizes.

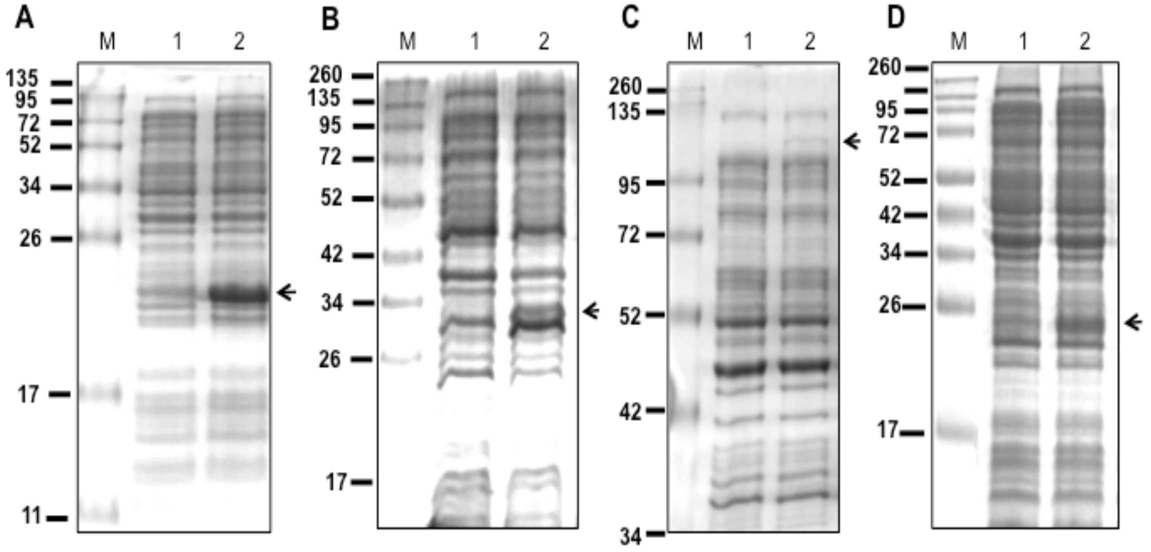

Figure S2. Expression of His6-tagged recombinant proteins. The whole-cell lysates were prepared from the cultures of *E. coli* BL21(DE3)pLysS strains that respectively expressed **A**. SigX-His6, **B.** MecA-His6, **C.** ClpC-His6, and **D.** ClpP-His6. The proteins in the cell lysates were separated in 10% SDS-PAGE gels and stained with Coomassie blue. M: molecular marker in Kilodalton; 1.The cell lysates prepared from the cultures without addition of IPTG; 2: The cell lysates prepared with addition of 0.5 mM IPTG for 2 hours. Arrows indicate expressed His6-tagged proteins with predicted molecular sizes.

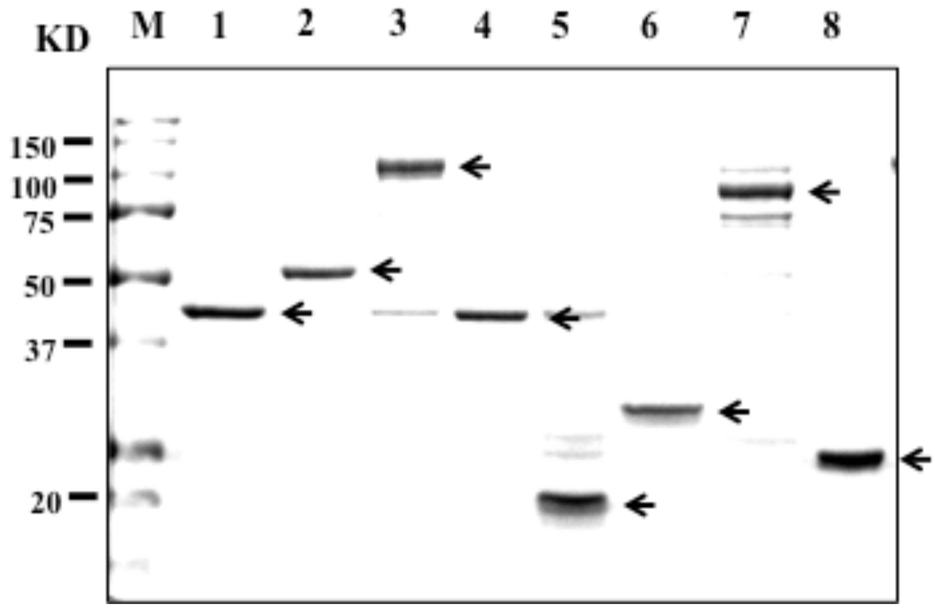

Figure S3. The identities of eight recombinant proteins purified from the whole-cell lysates of *E. coli* BL21(DE3)pLysS cultures, as described in Fig. S2-3. **Columns 1-4** indicate the GST-tagged recombinant proteins of SigX, MecA, ClpC and ClpP, which were purified with GenScript glutathione resins. **Columns 5-8** indicate the His6-tagged recombinant proteins of SigX, MecA, ClpC and ClpP, which were purified with GenScript Ni-NTA resins. All the proteins were dissolved on a 10% SDS-PAGE gel and stained with Coomassie blue. M: Molecular marker in Kilodalton (KD). Arrows indicate each recombinant protein with predicted molecular size.

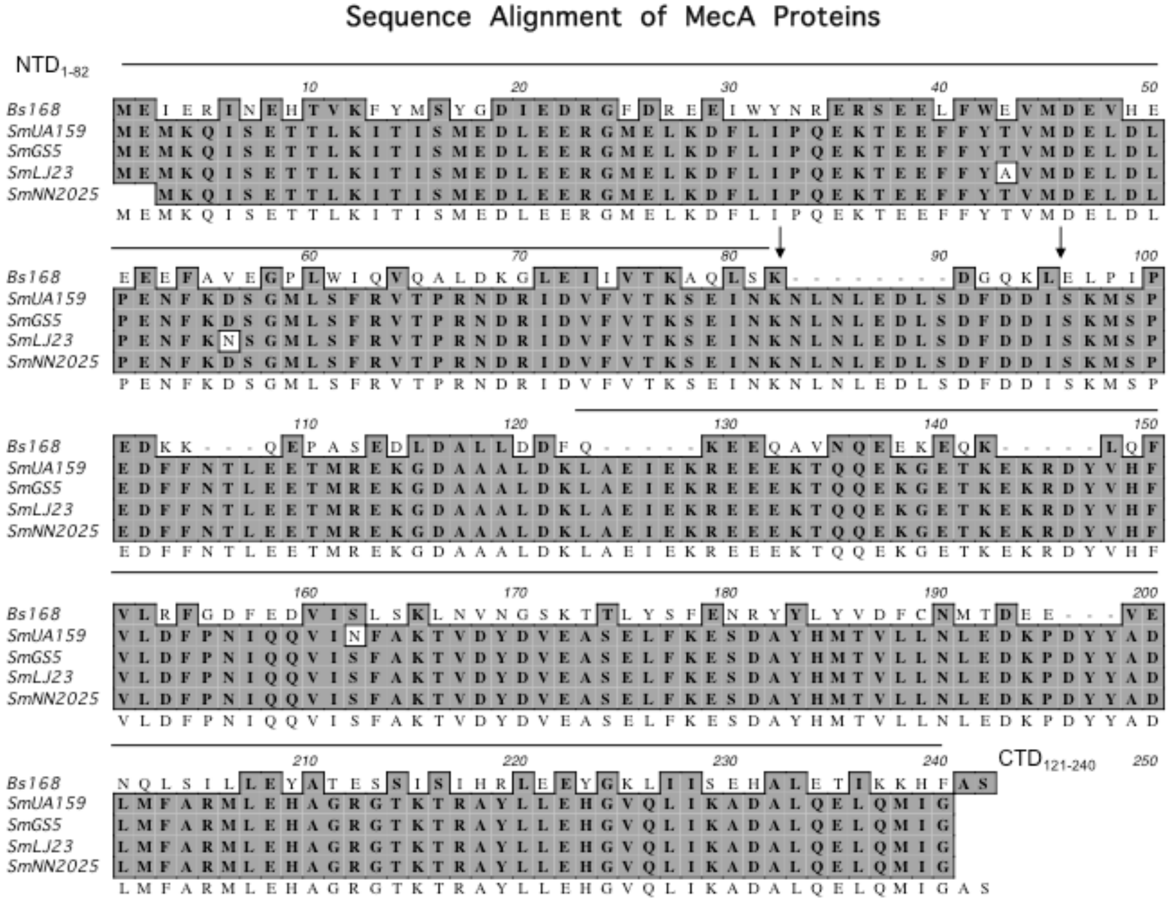

Figure S4. The sequence alignment of MecA proteins from *B. subtilis* 168 (Bs168) and *S. mutans* strains (SmUA159, SmGS-5, SmLJ23 and SmNN2025) were performed using the software of MacVector 9.0 ClusterW. The conservation of amino acid residues cross the strains is highlighted in the grey box. Black lines above the sequence alignment indicate the *N*-terminal domain (NTD_{1-82}) and the *C*-terminal domain ($CTD_{123-240}$) of MecA proteins. Vertical arrows indicate preferred cleavage sites of MecA by trypsin (K^{82}) and V8 protease (E^{93}) in *B. subtilis*. Gene bank accession numbers of all MecA proteins are: *B. subtilis* 168 (NP_389034.1), *S. mutans* UA159 (NP_72079.1), GS-5 (YP_006489516.1), LJ23 (YP_006252000.1) and NN2025 (YP_003485630.1).
